# Plasma Oxidized Albumin in Acute Ischemic Stroke Is Associated With Better Outcomes

**DOI:** 10.3389/fneur.2019.00709

**Published:** 2019-07-02

**Authors:** Leonard T. Rael, Jan Leonard, Kristin Salottolo, Raphael Bar-Or, Russell E. Bartt, Jeffrey C. Wagner, David Bar-Or

**Affiliations:** Trauma and Stroke Research Department, Swedish Medical Center, Englewood, CO, United States

**Keywords:** ischemic stroke, inflammation, oxidative stress, proteomics, albumin

## Abstract

**Introduction:** Plasma oxidized human serum albumin (OxHSA) is evidence of an active antioxidant mechanism as measured by oxidized species of HSA. CXCL-10 is a pro-inflammatory chemokine associated with ischemic conditions. Accordingly, we examined the relationship of admission OxHSA and CXCL-10 with discharge mRS in acute ischemic stroke (AIS).

**Methods:** Plasma samples and clinical data were collected prospectively at a Comprehensive Stroke Center. Admission biomarkers of oxidative stress, CXCL-10 and %OxHSA, were measured. We examined if CXCL-10 or %OxHSA correlated with age, admission NIHSS score, and discharge mRS score using Spearman's Rank correlation. Logistic regression was performed to identify independent predictors of a favorable discharge mRS (≤2).

**Results:** In 106 consecutive AIS patients, the median age was 73 (IQR 61–84), 47% were male, and the median admission NIHSS score was 11 (IQR 5–19). %OxHSA and CXCL-10 were significantly correlated (*r* = 0.23, *p* = 0.02). Both biomarkers were significantly correlated with age: %OxHSA (*r* = 0.44, *p* < 0.001) and CXCL-10 (*r* = 0.32, *p* = 0.001). Neither biomarker was correlated with admission NIHSS. There was a borderline significant correlation with discharge mRS and %OxHSA (*r* = −0.17, *p* = 0.08), where higher %OxHSA correlated with lower discharge mRS scores. For every 1% increase in %OxHSA, the odds of a favorable discharge mRS increased 11%. The odds of a favorable discharge mRS decreased 18% for every 1-point increase in the initial NIHSS.

**Conclusions:** OxHSA, the result of an oxidative environment and evidence of the strong antioxidant buffering capacity of HSA, correlated with CXCL-10 and discharge mRS, implying that strong antioxidant activity of albumin may confer better outcomes.

## Introduction

Acute ischemic stroke (AIS) is a serious health condition characterized by inadequate brain blood perfusion due to a blockage resulting in rapid loss of neurological function. Current treatment includes administration of tissue plasminogen activator (tPA) and/or intra-arterial therapy (i.e., thrombectomy) within a few hours of AIS. However, restoring blood flow can cause further damage to brain tissue. Reperfusion injury results in the reintroduction of oxygenated blood to the ischemic regions of the brain. This can induce biochemical pathways that produce reactive oxygen species (ROS) and pro-inflammatory mediators potentially leading to apoptosis and cellular necrosis (1). Oxidative stress markers include the presence of higher concentrations of lipid peroxides in plasma and urine within 24 h from the onset of AIS (2). Additional cellular targets include proteins which are susceptible to oxidative damage following reperfusion leading to immune system activation, neutrophil recruitment, and neurovascular inflammation (3). Indeed, protein oxidation is positively correlated with complications following AIS such as post-stroke depression resulting in increased rehabilitation times, disability/cognitive impairment, and mortality (4).

This study sought to correlate clinical outcomes in AIS patients with plasma biomarkers of oxidation and/or inflammation at admission. Two biomarkers of oxidative stress and inflammation were selected: human serum albumin (HSA) due to its unique antioxidant properties and high plasma concentrations (3.5–5.5 g/dL), and C-X-C motif chemokine ligand 10 (CXCL-10), a key chemoattractant involved in the recruitment of lymphocytes. In previous studies, we have shown that plasma oxidized HSA (OxHSA) levels are increased in severely traumatized patients specifically in traumatic brain injury (5). Additionally, OxHSA concentrations are positively correlated with an integrated measurement of oxidative stress, oxidation reduction potential (ORP). Finally, CXCL-10 plasma levels are significantly elevated in AIS patients (6).

## Materials and Methods

This prospective observational study was conducted at Swedish Medical Center (SMC), a high-volume, tertiary care urban Comprehensive Stroke Center. Study selection included adult patients (age ≥ 18 years) with signs and symptoms of stroke who were admitted over 2 years (1/1/2010–12/31/2011). This study was approved by the SMC HealthOne Institutional Review Board. Written informed consent was obtained from patients or a legally authorized representative.

Patients were selected for the analysis by meeting the following exclusion criteria: (1) discharge diagnosis of AIS; and (2) plasma sample collected on the day of admission and the subsequent day (hospital days 1 and 2).

Up to 10 ml of blood were collected no more than once daily during the patients' hospital stay. Venipuncture was carried out by a laboratory phlebotomist, and whole blood was collected in heparinized tubes for diagnostic purposes. Samples were obtained within 24 h of admission. Blood was processed to plasma, aliquoted, and stored at −80°C. Two laboratory biomarkers of oxidative stress and inflammation, the percent of oxidized human serum albumin (%OxHSA) and CXCL-10, were measured from the admission plasma samples. CXCL-10 in plasma was measured by ELISA (Thermo Fisher Scientific, Waltham, MA). %OxHSA was measured by liquid chromatography-mass spectrometry (LCMS) using a previously described method (5).

De-identified clinical data were collected from Neurobase™ (CDM, Evergreen, CO), the neurology department's stroke registry that contains a broad set of data prospectively collected by trained hospital personnel on stroke patients. The variables obtained included age, sex, race (white vs. other), independent pre-injury locomotion, initial and discharge National Institute of Health Stroke Scale (NIHSS) score, time from symptom onset to arrival, treatment strategy (IV tPA and/or IA therapy), discharge modified Rankin Scale (mRS) score, in-hospital mortality, and hospital length of stay (LOS).

### Statistical Analyses

The data were analyzed using SAS (version 9.4, Cary, NC). Statistical significance was set at *p* < 0.05. Spearman's Rank correlation and Pearson's Correlation were used to analyze whether the oxidative stress biomarkers (CXCL-10 or %OxHSA) were correlated with demographics and clinical assessments of stroke severity and inflammation (age, sex, race, pre-injury independent locomotion, admission and discharge NIHSS score, discharge mRS score, and hospital LOS). Differences by favorable discharge mRS score (≤ 2) were examined using Pearson or Fisher's exact chi-square test for categorical variables and the Wilcoxon two-sample test for continuous variables.

Logistic regression was performed to identify whether CXCL-10 or %OxHSA were independent predictors of a favorable discharge mRS (≤ 2), after adjusting for clinical covariates that were significant (*p* < 0.05) in either the correlation or univariate analysis (age and initial NIHSS). A subset analysis was performed in the patients who received IV tPA or IA therapy.

## Results

The analysis population included 106 consecutive patients with AIS. The median age was 73 years (IQR 61–84), 47% were male, and the median admission NIHSS score was 11 (IQR 5–19). The median %OxHSA was 39.5% (IQR 35.5–45.0%) and the median CXCL-10 was 167.2 pg/ml (IQR 94.5–281.3 pg/ml). Overall, 60 (57%) patients received treatment with IV tPA (*n* = 54) and/or IA therapy (*n* = 15). Approximately 38% of the patients had a favorable (≤ 2) mRS at discharge and 10% of the patients died while in the hospital (Table 1).

**Table 1:** Correlation coefficients and *p*-values.

**Spearman or Pearson correlation coefficient**	**CXCL-10**	***p*-value**	**%OxHSA**	***p*-value**
CXCL-10			0.23	**0.02**
%OxHSA	0.23	**0.02**		
Age	0.32	**0.001**	0.44	**<0.001**
Sex^*^	−0.003	0.97	0.13	0.19
Race^*^	0.07	0.45	−0.12	0.23
IV tPA or IA therapy^*^	0.07	0.51	0.08	0.39
Independent pre-injury locomotion^*^	0.02	0.85	0.13	0.18
Initial NIHSS	−0.03	0.75	−0.11	0.25
Discharge NIHSS	0.04	0.73	−0.05	0.65
Discharge mRS	0.02	0.83	−0.17	***0.08***
Length of stay	−0.15	0.12	0.05	0.64

IV, intravenous; IA, intra-arterial; HSA, human serum albumin; NIHSS, National Institutes of Health Stroke Scale.

* *Pearson Correlation Coefficient. Bold signifies statistical significance. Italics signifies approaching statistical significance*.

There were no differences observed in patient's age, sex, race, treatment strategy, or independent pre-injury locomotion by favorable discharge mRS score. Patients with a favorable discharge mRS score had a significantly lower initial NIHSS score and a significantly higher %OxHSA. Patients with a favorable discharge mRS score also had a significantly lower NIHSS score at discharge and a shorter hospital LOS (Supplementary Table 1).

The %OxHSA and CXCL-10 were significantly correlated (*r* = 0.23, *p* = 0.02) with each other (Table 1). These two biomarkers were also significantly correlated with age. Neither biomarker was correlated with sex, race, treatment strategy, independent pre-injury locomotion, admission NIHSS, or LOS. There was a borderline significant correlation between the discharge mRS and %OxHSA (*r* = −0.17, *p* = 0.08), where a higher %OxHSA was correlated with lower mRS scores (better outcomes).

In logistic regression analysis, %OxHSA (AOR 1.11, *p* = 0.01) and initial NIHSS score (AOR 0.82, *p* < 0.001) were independently associated with a favorable discharge mRS, controlling for age and CXCL-10 (Table 2). These results were also observed in the subset of patients who received IV tPA or IA therapy (Table 2).

**Table 2:** Multivariate predictors of a favorable discharge mRS ≤ 2.

**Covariates**	**AOR (95% CI)**	***p*-value**
**Overall (*****n*** **=** **106)**, ***c*** **=** **0.86**		
Age	0.99 (0.95–1.02)	0.40
Initial NIHSS	0.82 (0.76–0.90)	**<0.001**
% OxHSA	1.11 (1.03–1.20)	**0.01**
CXCL-10	1.00 (0.998–1.002)	0.86
**IV tPA or IA therapy (*****n*** **=** **60)**, ***c*** **=** **0.90**		
Age	0.97 (0.93–1.02)	0.24
Initial NIHSS	0.78 (0.67–0.90)	**<0.001**
% OxHSA	1.12 (1.001–1.25)	**0.048**
CXCL-10	1.00 (0.998–1.002)	0.87

*AOR, adjusted odds ratio; CI, confidence interval; c = c-statistic; HSA, human serum albumin; NIHSS, National Institutes of Health Stroke Scale; IV, intravenous; IA, intra-arterial. Bold signifies statistical significance*.

## Discussion

The results of the logistic regression model demonstrate that the odds of a favorable outcome increase as the %OxHSA increase. This finding, which may seem counterintuitive because higher %OxHSA is associated with oxidative stress, may be evidence of greater antioxidant buffering capacity of HSA. As expected, the odds of a favorable outcome increased as the NIHSS score decreased (i.e., less stroke severity). Age and CXCL-10 levels were not independently associated with a favorable outcome.

The antioxidant capabilities of HSA are well documented with most of its antioxidant capacity arising from the free sulfhydryl (SH-) group of a cysteine residue in position 34 which accounts for 90% of the free SH- groups in circulation (7). This cysteine residue reduces disulfide bonds in circulation resulting in the regeneration of free sulfhydryl groups which can further participate in antioxidant-type reactions (8). In AIS, OxHSA was also discovered in cerebrospinal fluid indicating that HSA is involved in antioxidant-type reactions within the neurological milieu (9). Therefore, the question arises if HSA administration to AIS patients is beneficial or not. Regarding HSA therapy, the literature provides mixed results with some studies demonstrating improved neurological function, reduction in infarct volume, and decreased brain swelling up to 4 h after onset of ischemia (10). However, a Phase 3 clinical trial with a placebo control arm showed no clinical benefit of HSA administration 5 h after the onset of ischemia (11). Of note, the timing of HSA administration is important and appears to follow the critical time frame of tPA therapy in that administration within 4 h is necessary for maximum efficacy. Also, the source and age of the administered HSA is critical since commercially available HSA accumulates OxHSA over time resulting in the gradual loss of the antioxidant capacity of HSA (7). Therefore, the mixed clinical findings could be partially explained by the age of the administered HSA with older lots containing more OxHSA resulting in undesirable outcomes due to less antioxidant capacity of HSA.

CXCL-10 and OxHSA positively correlated with each other demonstrating that these biomarkers are indicative of an active inflammatory process occurring. Additionally, both biomarkers positively correlated with age which is not surprising since it is well known that the aging process results in the progressive loss of tissue and organ function over time due to the gradual accumulation of oxidized macromolecules (12). CXCL-10 was not predictive of stroke severity based on mRS score in our study. CXCL-10 is a small chemokine that functions as a chemoattractant for many immune cells such as lymphocytes and is a possible biomarker in cardiovascular disease (13). More importantly, serum CXCL-10 levels are significantly elevated in patients with AIS (6).

Limitations of this study include a small sample size of 106 AIS patients. A larger study of AIS patients with varying degrees of stroke severity based on NIHSS and mRS scores is warranted to determine if the conclusions of this study are valid. Additionally, the timing of sample collection in this study covers a wide range from 0 to 24 h post-admission. It is possible that the peak time for assessing reperfusion injury was missed in some patients. Therefore, a future study should collect samples from AIS patients during a smaller time window to ensure that the results are consistent from patient to patient. Our group is currently conducting a prospective study on AIS patients and collecting plasma samples before (ischemic phase) and after (reperfusion phase) clot removal. We believe that the results from this current study will provide valuable insight into the relevant biochemical pathways occurring in AIS patients.

We believe that the findings in our study could provide insight into some of the biochemical pathways involved after AIS. Every human possesses natural antioxidant defenses such as ascorbate, albumin, and enzymes (i.e., superoxide dismutase). Therefore, initial levels of these defenses are potentially important in determining outcomes in diseases characterized by oxidative stress such as AIS. The ability to quench free radicals by scavenger molecules such as HSA is crucial in preventing the oxidation of key macromolecules (lipid bilayers, DNA, critical enzymes, etc.). These results suggest plasma CXCL-10 represents a general marker of inflammation and oxidative stress and therefore is not predictive of loss of function or disability in AIS. However, OxHSA may be predictive of clinical outcomes following AIS based on the results of this study.

## Conclusions

In our study, higher amounts of OxHSA correlated with better outcomes in AIS patients based on mRS scores. This suggests that more OxHSA translates into better protection from the amount of ROS produced by AIS patients. These findings agree with previous findings which suggest relatively high HSA levels in AIS patients decreases the risk of poor outcomes based on mRS scores (14). Future studies should focus on other markers of oxidation using LCMS proteomics analysis of plasma proteins as well as measuring the overall oxidative stress and antioxidant capacity of plasma samples obtained from AIS patients. This information could be useful in determining the optimal timing and type of combination treatment (IV tPA or IA therapy with an antioxidant such as inhibitors of ROS generating enzymes, free radical scavengers, promoters of ROS degrading enzymes, etc.) which have thus far reported mixed results (15, 16).

## Data Availability

The datasets generated for this study are available on request to the corresponding author.

## Ethics Statement

This study was carried out in accordance with the recommendations of the SMC HealthOne Institutional Review Board with written informed consent from all subjects. All subjects gave written informed consent in accordance with the Declaration of Helsinki. The protocol was approved by the SMC HealthOne Institutional Review Board.

## Author Contributions

LR, JL, and KS participated in the acquisition, analysis, and interpretation of data, drafting the article, and revised it critically for important intellectual content. RB-O participated in the acquisition of data, drafting the article, and revised it critically for important intellectual content. RB and JW participated in drafting the article and revised it critically for important intellectual content. DB-O made contributions to conception and design, participated in drafting the article, and revised it critically for important intellectual content.

### Conflict of Interest Statement

The authors declare that the research was conducted in the absence of any commercial or financial relationships that could be construed as a potential conflict of interest.
