# Supplementary material for: Plasma Oxidized Albumin in Acute Ischemic Stroke Is Associated With Better Outcomes
Source: Front Neurol. 2019 Jul 2;10:709. doi: 10.3389/fneur.2019.00709 (PMC6614430; doi:10.3389/fneur.2019.00709)
Supplement: Supplementary file 1 [file Table_1.DOCX]

Supplementary Material

# Supplementary Table

| **Supplementary Table 1. Characteristics of the Study Population Overall and by Discharge mRS.** | | | | |
| --- | --- | --- | --- | --- |
|  | **n (%)**  **(n=106)** | **Discharge mRS ≤ 2**  **(n=40)** | **Discharge mRS ≥ 3**  **(n=66)** | **p-value** |
| Age, median (IQR) | 73 (61-84) | 80.5 (57-85) | 72 (62-81) | 0.34 |
| Male | 50 (47.2%) | 15 (37.5%) | 35 (53.0%) | 0.12 |
| Race  White  Other | 89 (84.0%)  17 (16.0%) | 34 (85.0%)  6 (15.0%) | 55 (83.3%)  11 (16.7%) | 0.82 |
| Independent Pre-Injury Locomotion | 96 (90.6%) | 34 (85.0%) | 62 (93.9%) | 0.17 |
| Initial NIHSS score, median (IQR)^*^  Minor (<5)  Moderate (5-15)  Moderate/Severe (16-20)  Severe (≥21)  NIHSS score < 17 | 11 (5-19)  22 (21.4%)  42 (40.8%)  19 (18.5%)  20 (19.4%)  66 (64.1%) | 6 (3-9)  16 (43.2%)  19 (51.4%)  2 (5.4%)  0 (0%)  35 (94.6%) | 17 (8-21)  6 (9.1%)  23 (34.9%)  17 (25.8%)  20 (30.3%)  31 (47.0%) | **<0.001**  **<0.001**  **<0.001** |
| CXCL-10, median (IQR) | 167.2 (94.5-281.3) | 145.1 (105.4-263.2) | 185.0 (90.6-285.7) | 0.95 |
| %OxHSA, median (IQR) | 39.5% (35.5%-45.0%) | 43.2% (38.3%-48.4%) | 38.1% (34.5%-43.8%) | **0.01** |
| Discharge NIHSS score, median (IQR)^†^ | 4 (1-9) | 1 (0-2) | 4.5 (4-14) | **<0.001** |
| In-hospital Death | 11 (10.4%) | 0 (0%) | 11 (16.7%) | **0.01** |
| Hospital Length of Stay, days, median (IQR)^‡^ | 5 (3-9) | 3 (2-5) | 7 (4-10.5) | **<0.001** |
| ^*^Missing values for 3 patients; ^†^Missing values for 17 patients; ^‡^Missing values for 2 patients | | | | |
